# Alternatively Activated (M2) Macrophage Phenotype Is Inducible by Endothelin-1 in Cultured Human Macrophages

**DOI:** 10.1371/journal.pone.0166433

**Published:** 2016-11-15

**Authors:** Stefano Soldano, Carmen Pizzorni, Sabrina Paolino, Amelia Chiara Trombetta, Paola Montagna, Renata Brizzolara, Barbara Ruaro, Alberto Sulli, Maurizio Cutolo

**Affiliations:** Research Laboratory and Academic Division of Clinical Rheumatology, Department of Internal Medicine, University of Genova, Genoa, Italy; Universitatsklinikum Freiburg, GERMANY

## Abstract

**Background:**

Alternatively activated (M2) macrophages are phenotypically characterized by the expression of specific markers, mainly macrophage scavenger receptors (CD204 and CD163) and mannose receptor-1 (CD206), and participate in the fibrotic process by over-producing pro-fibrotic molecules, such as transforming growth factor-beta1 (TGFbeta1) and metalloproteinase (MMP)-9. Endothelin-1 (ET-1) is implicated in the fibrotic process, exerting its pro-fibrotic effects through the interaction with its receptors (ET_A_ and ET_B_). The study investigated the possible role of ET-1 in inducing the transition from cultured human macrophages into M2 cells.

**Methods:**

Cultured human monocytes (THP-1 cell line) were activated into macrophages (M0 macrophages) with phorbol myristate acetate and subsequently maintained in growth medium (M0-controls) or treated with either ET-1 (100nM) or interleukin-4 (IL-4, 10ng/mL, M2 inducer) for 72 hours. Similarly, primary cultures of human peripheral blood monocyte (PBM)-derived macrophages obtained from healthy subjects, were maintained in growth medium (untreated cells) or treated with ET-1 or IL-4 for 6 days. Both M0 and PBM-derived macrophages were pre-treated with ET receptor antagonist (ET_A/B_RA, bosentan 10^-5^M) for 1 hour before ET-1 stimulation. Protein and gene expression of CD204, CD206, CD163, TGFbeta1 were analysed by immunocytochemistry, Western blotting and quantitative real time polymerase chain reaction (qRT-PCR). Gene expression of interleukin(IL)-10 and macrophage derived chemokine (CCL-22) was evaluated by qRT-PCR. MMP-9 production was investigated by gel zymography.

**Results:**

ET-1 significantly increased the expression of M2 phenotype markers CD204, CD206, CD163, IL-10 and CCL-22, and the production of MMP-9 in both cultures of M0 and PBM-derived macrophages compared to M0-controls and untreated cells. In cultured PBM-derived macrophages, ET-1 increased TGFbeta1 protein and gene expression compared to untreated cells. The ET-1-mediated effects were contrasted by ET_A/B_RA treatment in both cultured cell types.

**Conclusion:**

ET-1 seems to induce the M2 phenotype in cultured human macrophages, a process apparently contrasted by the action of the ET_A/B_RA, suggesting possible clinical implications in those fibrotic diseases characterized by increased ET-1 concentrations, such as systemic sclerosis but also type 2 diabetes.

## Introduction

Macrophages are key mediators of innate and adaptive immunity and they contribute to homeostasis and tissue remodelling [1–3]. Local microenvironment drives macrophages to acquire distinct functional and morphological properties and to polarize into classically activated (M1) or alternatively activated (M2) macrophages [3,4]. The M1 macrophages show a pro-inflammatory phenotype, contributing to the intensive inflammation and tissue damage or even predisposing to neoplastic transformation [4,5]. The M2 macrophages are involved in wound healing by their anti-inflammatory properties [1–4]. Recently, M2 macrophages were found to show a pro-fibrotic phenotype being able to induce fibrosis as well as exacerbate the allergic process [4,5]. Although these macrophage subsets express distinct and specific phenotype markers, they are considered extremes of a continuum of functional states [3,6].

The M2 macrophages are activated by T-helper-2 cytokines (i.e. IL-4 and IL-13) and they are characterized by the expression of specific phenotype markers, primarily scavenger receptors-1 class A (CD204), mannose receptor-1 (CD206) and hemoglobin scavenger receptor (CD163) [1,4]. These macrophages are also characterized by the production of specific cytokines and chemokines, such as interleukin-10 (IL-10) and macrophage derived chemokine (MDC or CCL-22), pro-fibrotic metalloproteases (i.e. MMP-9) and transforming growth factor-beta1 (TGFbeta1) [1,3]. The release of these molecules contributes to myofibroblast activation and the deposition of extracellular matrix components [2,7,8].

Recently, CD14^bright^CD204^+^/CD163^+^M2 macrophages were shown to be increased in the peripheral blood and affected skin of systemic sclerosis (SSc) patients, thus indicating their possible role in the pathogenesis of the disease, as well as in the development of dermal fibrosis [9,10].

Endothelin-1 (ET-1) is a pro-fibrotic molecule mainly secreted by endothelial cells and its plasma levels are highly increased in patients affected by SSc and type 2 diabetes, contributing to the fibrotic process [11,12]. ET-1 exerts pro-fibrotic effects through the binding to its receptors (ET_A_ and ET_B_), which are expressed on several cell types including endothelial cells, fibroblasts and macrophages [13–15].

Based on these observations, the aim of the study was to investigate the ability of ET-1 to induce the transition of cultured human macrophages towards an M2 phenotype, evaluating the expression of specific markers and the over-production of pro-fibrotic molecules. Moreover, the action of a dual ET receptor antagonist (ET_A/B_RA, bosentan) in contrasting the M2 phenotype induction mediated by ET-1 was also investigated.

## Materials and Methods

### Cell Cultures and Treatments

Human monocyte cells (THP-1 cell line, 1x10^6^ cells/mL, accession number ICLC HTL97014, Cell bank Interlab Cell Line Collection, Genoa, Italy) were plated in cell culture dishes and stimulated with phorbol myristate acetate (PMA, 50ng/mL, Sigma-Aldrich, Italy) for 4 hours to induce their differentiation into macrophages (M0 macrophages), in accordance with several *in vitro* studies [16–18]. Once differentiated, PMA was removed and some M0 macrophages were treated for 72 hours with ET-1 (100nM, Enzo Life Science, UK) or pre-treated for 1 hour with ET_A/B_RA (bosentan, 10^-5^M, Actelion Pharmaceutics, Switzerland) before being stimulated with ET-1. The lyophilized ET-1 molecule was solved in sterile distilled water. All treatments were made in RPMI at 5% fetal bovine serum (FBS), 1% penicillin-streptomycin and 1% L-glutamine (Euroclone, Italy). Cultured M0 macrophages maintained in RPMI at 5% FBS were used as untreated control cells (M0-controls). Other cultured M0 macrophages were treated for 72 hours with IL-4 (10ng/mL, BioVision, CA, USA), as an inducer of the M2 phenotype, thus representing a positive control of the M2 macrophage polarization [1–4,19]. Preliminary time-dependent experiments about treating cultured M0 macrophages with ET-1 for 24, 48 and 72 hours were carried out in order to investigate its ability to induce the expression of the M2 macrophage phenotype markers. Preliminary results showed that ET-1 induced the expression of CD204, CD206 and CD163 after 48 and 72 hours, whereas no effects were observed after 24 hours (S1 Fig).

Based on these preliminary data, the effects of ET-1 in inducing the expression of all the M2 macrophage markers tested were investigated at 72 hours on cultured M0 macrophages.

At the same time, human monocytes were isolated from the peripheral blood mononuclear cells (PBMCs) of 6 voluntary healthy subjects (mean age 42±10 years) using Ficoll-density gradient and a monocyte isolation kit (Miltenyi Biotech, Italy). Human monocytes were analysed by Flow cytometry using an anti-CD14-PE-Vio770 antibody (clone TÜK4, Miltenyi Biotech).

Once isolated, cells were plated into tissue culture dishes (1.5x10^6^ cells/mL) and maintained in RPMI at 10% of FBS for 24 hours. Some cultured human monocytes were treated for 3 and 6 days with either ET-1 (100nM) or IL-4 (10ng/mL) in order to induce their differentiation into M2 macrophages. Other cultured cells were pre-treated for 1 hour with ET_A/B_RA (10^-5^M) and then stimulated with ET-1 for 3 and 6 days, in accordance with several *in vitro* studies [20,21]. Cultured human monocytes maintained in RPMI at 10% of FBS were used as untreated cells.

Six independent *in vitro* experiments were performed on both cultured cell types, respectively.

### Immunocytochemistry

THP-1 cells and human monocytes were treated as described in the “Cell cultures and treatment” paragraph. At the end of treatments, cells were fixed in 2% paraphormaldeide and incubated with primary antibodies to human CD68 (clone KP1, dilution 1:200, macrophage marker), CD204 (clone E5, dilution 1:200) and CD206 (clone 6A598, dilution 1:150) (Santa Cruz Biotechnology, CA, USA). Linked antibodies were detected by biotin-conjugated secondary antibody and subsequently with horseradish-peroxydase (HRP)-streptavidin complex (Vector Laboratories, CA, USA). The 3,3’-diaminobenzidine was used to detect the expression of the investigated proteins. In each experimental condition, the detection of protein expression was performed evaluating the same number of cells by light microscopy (magnification 40X) (Leica, UK).

### Western Blotting

Total proteins were isolated by NucleoSpin RNA/protein (Macherey-Nagel, Germany) and quantified by the Bradford method. For every condition, 0.035mg of protein were separated by electrophoresis on 4–16% tris-glycine gel and transferred onto Hybond-C-nitrocellulose membrane (Life Technologies Ltd, UK).

After 1 hour in blocking solution -phosphate buffer saline (PBS) 1x, 0.1% triton-X and 5% non-fat powdered milk-, membranes were incubated overnight at 4°C with primary antibodies anti-human CD204 (clone E5, dilution 1:150, Santa Cruz Biotechnology), CD206 (clone 2A6A10, dilution 1:500, Proteintech, USA), CD163 (clone GHI/61, dilution 1:100, Novus Biological, Littleton, USA) and TGFbeta1 (dilution 1:2,000, GeneTex, Texas, USA).

Membranes were subsequently incubated for 1 hour with secondary antibody (dilution 1:2,000 Cell Signaling Technology, MA, USA).

To confirm similar loading of gels and the efficiency in the electrophoretic transfer, membranes were also incubated with primary HRP-conjugated antibody to human beta-actin (clone C4, dilution 1:5,000 Santa Cruz Biotechnology).

Protein synthesis was analysed by enhanced chemiluminescence system (Luminata Crescendo, Millipore, Germany) and the densitometric analysis was performed by UVITEC analysis software (UVITEC, UK). In both cultured cell types, for each experimental condition, the values of CD204, CD206, CD163 and TGFbeta1 synthesis were normalized to those of the corresponding beta-actin. The resulting values of each treatment were subsequently normalized to that of M0-controls or untreated cells respectively (taken as unit value by definition), in order to obtain the level of protein synthesis.

### Quantitative Real Time Polymerase Chain Reaction (qRT-PCR)

Total RNA was extracted with NucleoSpin RNA/protein (Macherey-Nagel) and quantified by nanodrop (Thermo Scientific, USA), which also evaluated RNA integrity, in accordance with the manufacturer’s protocol. For each experimental condition, first-strand cDNA was synthesized from 0.001mg of total RNA using QuantiTect Reverse Transcription Kit (Qiagen, Italy).

Primers for human CD204 (NM_002445), CD206 (NM_002438), CD163 (NM_004244), IL-10 (NM_000572), CCL-22 (NM_002990), TGFbeta1 (NM_000660) and beta-actin (NM_001101, housekeeping gene) were supplied by Primerdesign (Primerdesign, UK). In both cultured cell types, gene expression values were calculated using the comparative ΔΔCT method and they correspond to the expression level (fold increase) of the target gene derived from each treatment and normalized to that of M0-controls or untreated cells respectively (taken as unit value by definition) [22]. In all qRT-PCR, the melting curve was performed to confirm the specificity of the SYBR green assay.

### Gel Zymography

The conditioned medium was collected and the total amount of protein quantified by Bradford method. For every condition, 0.035mg of proteins were separated by electrophoresis on 10% tris-glycine gel containing gelatin (1mg/mL, Sigma-Aldrich). The gel was washed in PBS1x and 0.1% triton-X for 1 hour and then incubated overnight at 37°C in a solution containing CaCl_2_ 10mM, NaCl 200nM, TRIS 40mM pH7.5. The gel was coloured with 0.2% brilliant blue (Sigma-Aldrich) in a solution of 50% methanol and 10% acetone and subsequently incubated in a de-stained solution (50% methanol, 10% acetone). The MMP-9 production was quantified by UVITEC analysis software (UVITEC).

After densitometric analysis, in both cultured cell types, the values of each treatment were normalized to that of M0-controls or untreated cells, respectively (taken as unit value by definition), obtaining the increased production of MMP-9.

### Statistical Analysis

The statistical evaluation was carried out by GraphPad Prism 5 analysis software using the Mann-Whitney-*U* non-parametric test. Any p-value lower than 0.05 was considered as statistically significant. Results were indicated as mean±standard deviation (SD).

## Results

### Effects of ET-1, ET_A/B_RA and IL-4 on CD68, CD204 and CD206 Expression in Cultured Macrophages

**Fig 1:**
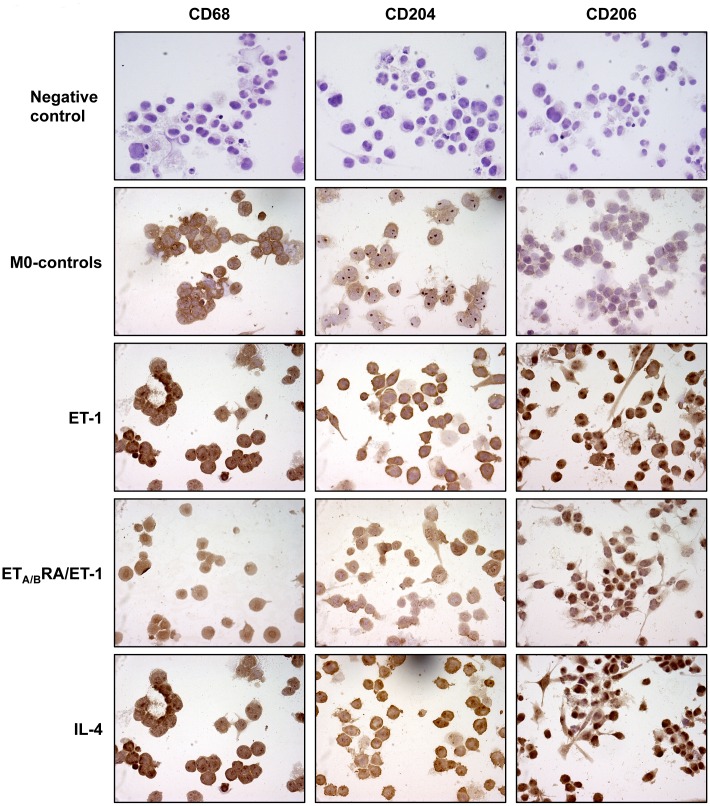
Evaluation of CD68, CD204 and CD206 expression in cultured THP-1-derived macrophages. Immunocytochemistry of CD68 (marker of macrophage activation), CD204 and CD206 protein expression in cultured THP-1-derived macrophages (M0 macrophages) treated for 72 hours with ET-1 (100nM) and IL-4 (10ng/mL) alone, or pre-treated with ET_A/B_RA (bosentan, 10^-5^M) for 1 hour before being stimulated with ET-1. Cultured M0 macrophages maintained for 72 hours in RPMI at 5% of FBS were used as controls (M0-controls). Immunocytochemistry was performed on four independent *in vitro* experiments. The detection of protein expression was performed evaluating the same number of cells by light microscopy (magnification 40X).

As observed by immunocytochemistry, in cultured THP-1 the stimulation with PMA induced cell adhesion and the expression of CD68 (macrophage marker), supporting their differentiation into M0 macrophages (Fig 1).

This result was in accordance with several *in vitro* studies, indicating THP-1-derived macrophages as a well-characterized model for studying macrophage polarization [16,17,23–25].

In cultured M0 macrophages, ET-1 increased the protein expression of CD204 and CD206 compared to M0-controls after 72 hours of treatment (Fig 1). Of note, the increase in the protein expression of these M2 phenotype markers induced by ET-1 was contrasted by the action of ET_A/B_RA (Fig 1).

The ability of ET-1 to induce M2 polarization was evaluated also in primary cultures of human monocyte-derived macrophages.

**Fig 2:**
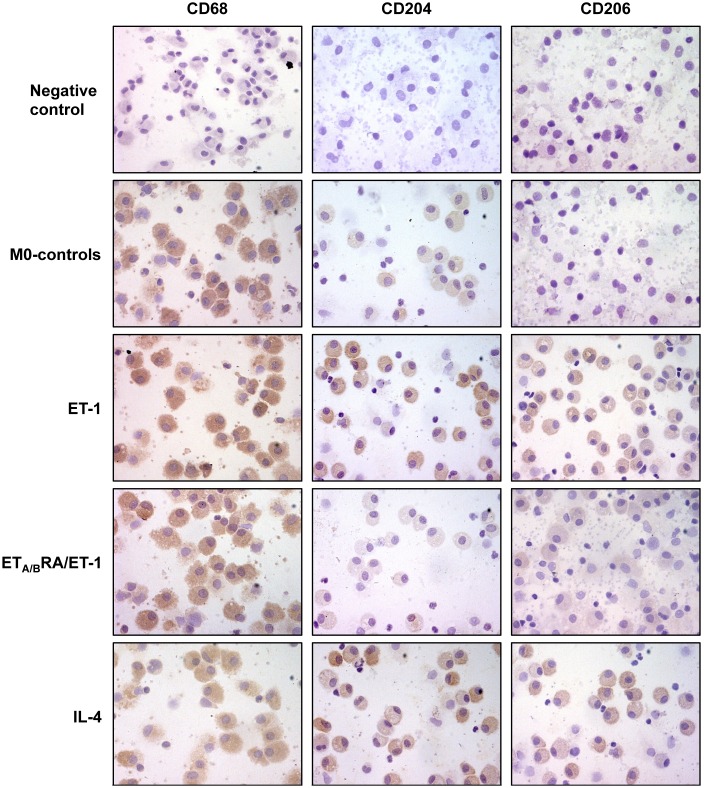
Evaluation of CD68, CD204 and CD206 expression in cultured human monocyte-derived macrophages. Immunocytochemistry of CD68 (marker of macrophage activation), CD204 and CD206 protein expression in cultured human monocyte-derived macrophages treated for 6 days with ET-1 (100nM) and IL-4 (10ng/mL) alone, or pre-treated with ET_A/B_RA (bosentan, 10^-5^M) for 1 hour before being stimulated with ET-1. Cultured human monocyte-derived macrophages maintained for 6 days in RPMI at 10% of FBS were used as untreated cells. Immunocytochemistry was performed on four independent *in vitro* experiments. The detection of protein expression was performed evaluating the same number of cells by light microscopy (magnification 40X).

Preliminary results showed that both untreated and treated cells did not express CD68 after 3 days of culture, indicating their incomplete differentiation into macrophages (data not shown). On the contrary, after 6 days, cultured cells were able to express CD68, confirming their differentiation into activated macrophages (Fig 2).

Based on these results, in cultured human monocyte-derived macrophages the effects of ET-1 and ET_A/B_RA were evaluated at 6 days of treatment.

In these primary cultures, ET-1 induced the increase in the protein expression of CD204 and CD206 compared to untreated cells (Fig 2). Of note, once again ET_A/B_RA antagonized this increased expression of both M2 phenotype markers induced by ET-1 (Fig 2).

In both cultured M0 and human monocyte-derived macrophages, IL-4 induced the expression of CD204 and CD206, confirming its role as an inducer of M2 polarization (Figs 1 and 2).

### Effects of ET-1, ET_A/B_RA and IL-4 on Protein Synthesis of M2 Phenotype Markers in Cultured Macrophages

To confirm the results observed by immunocytochemistry and to determine the increase in protein synthesis of M2 phenotype markers, Western blotting was performed on both cell types.

**Fig 3:**
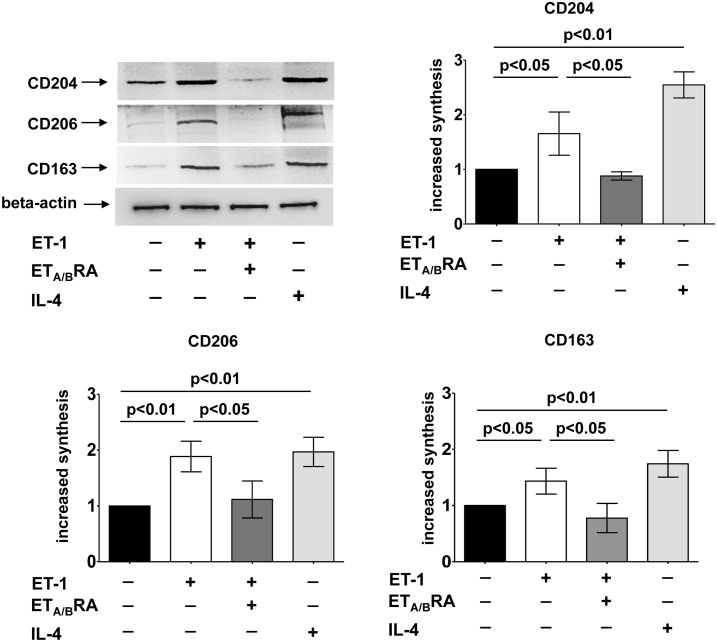
Evaluation of protein synthesis of M2 phenotype markers in cultured THP-1-derived macrophages. Western blotting and related densitometric analysis of CD204, CD206 and CD163 protein synthesis in cultured THP-1-derived macrophages (M0 macrophages) treated for 72 hours with ET-1 (100nM) and IL-4 (10ng/mL) alone, or pre-treated with ET_A/B_RA (bosentan, 10^-5^M) for 1 hour before being stimulated with ET-1. Cultured M0 macrophages maintained for 72 hours in RPMI at 5% of FBS were used as controls (M0-controls). Western blotting was performed on six independent *in vitro* experiments. The data of CD204, CD206 and CD163 protein synthesis are shown as mean±SD and indicated as increase in protein synthesis.

In cultured M0 macrophages, ET-1 induced a significant over-production of CD204, CD206 and CD163 compared to M0-controls (p<0.05; p<0.01; p<0.05) (Fig 3).

The ability of ET-1 to increase the protein synthesis of these M2 phenotype markers was found to be similar to that induced by IL-4 (p<0.01 vs. M0-controls, for all proteins) (Fig 3). Of note, ET_A/B_RA significantly contrasted the increased protein expression of these M2 markers (p<0.05 vs ET-1 treated cells for all proteins) (Fig 3).

**Fig 4:**
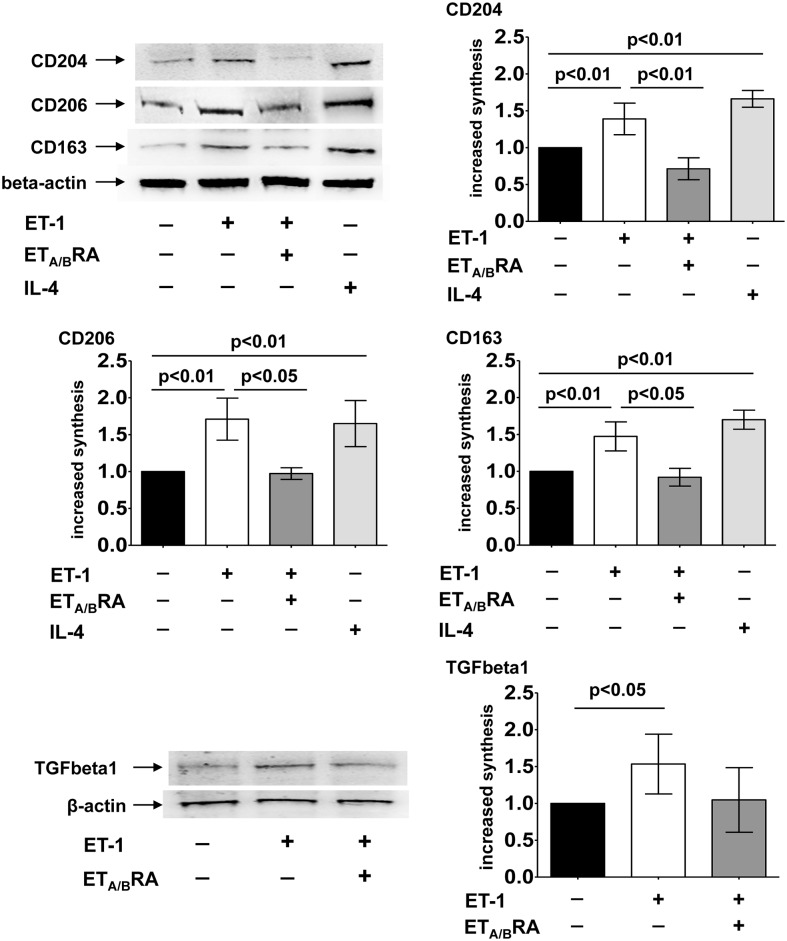
Evaluation of protein synthesis of M2 phenotype markers and TGFbeta1 in cultured human monocyte-derived macrophages. Western blotting and related densitometric analysis of CD204, CD206, CD163 and TGFbeta1 protein synthesis in cultured human monocyte-derived macrophages treated for 6 days with ET-1 (100nM) and IL-4 (10ng/mL) alone, or pre-treated with ET_A/B_RA (bosentan, 10^-5^M) for 1 hour before being stimulated with ET-1. Cultured human monocyte-derived macrophages maintained for 6 days in RPMI at 10% of FBS were used as untreated cells. Western blotting was performed on six independent *in vitro* experiments. The data of CD204, CD206, CD163 and TGFbeta1 protein synthesis are shown as mean±SD and indicated as increase in protein synthesis.

In cultured human monocyte-derived macrophages, ET-1 significantly induced the over-production of CD204, CD206 and CD163 protein synthesis compared to untreated cells (p<0.01 for all proteins), showing effects similar to those of IL-4 (p<0.01 vs untreated cells, for all proteins) (Fig 4).

In primary cultures of human monocyte-derived macrophages, the protein synthesis of TGFbeta1 was also investigated. Of note, ET-1 induced a significant increase in TGFbeta1 synthesis compared to untreated cells (p<0.05) (Fig 4).

In these cells, the ability of ET-1 to induce the protein expression of M2 phenotype markers was significantly antagonized by ET_A/B_RA (p<0.01 for CD204; p<0.05 for CD206 and CD163 vs. ET-1 treated cells), which also contrasted the ET-1-induced increase in TGFbeta1 protein expression (non significantly) (Fig 4).

### Effects of ET-1, ET_A/B_RA and IL-4 on the Gene Expression of M2 Phenotype Markers in Cultured Macrophages

**Fig 5:**
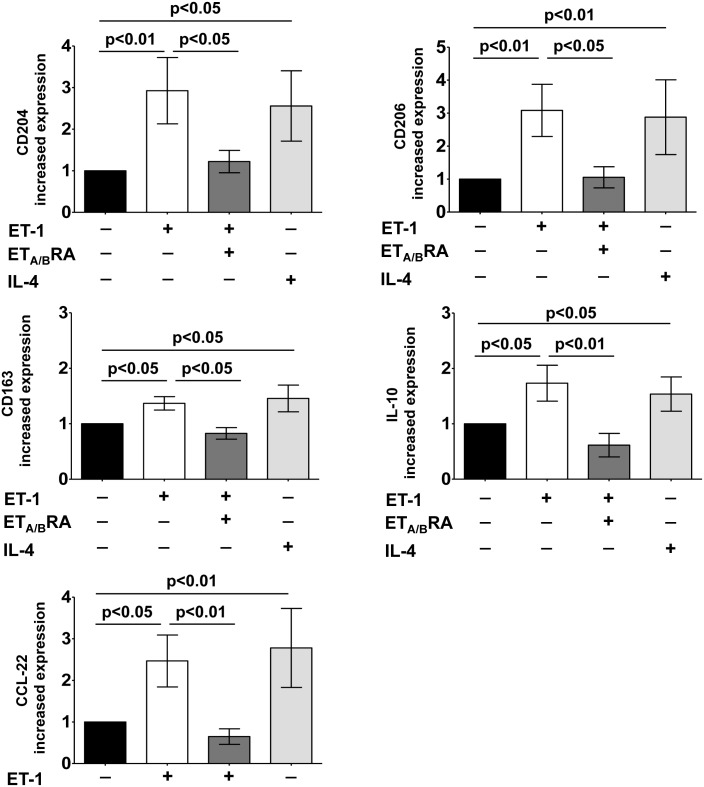
Evaluation of gene expression of M2 macrophage phenotype markers in cultured THP-1-derived macrophages. Quantitative real time polymerase chain reaction (qRT-PCR) of CD204, CD206, CD163, IL-10 and CCL-22 gene expression in cultured THP-1-derived macrophages (M0 macrophages) treated for 72 hours with ET-1 (100nM) and IL-4 (10ng/mL) alone, or pre-treated with ET_A/B_RA (bosentan, 10^-5^M) for 1 hour before being stimulated with ET-1. Cultured M0 macrophages maintained for 72 hours in RPMI at 5% of FBS were used as controls (M0-controls). The qRT-PCR was performed on six independent *in vitro* experiments and the data of CD204, CD206, CD163, IL-10 and CCL-22 gene expression are shown as mean±SD and indicated as increase in gene expression.

In cultured M0 macrophages, ET-1 induced a significant increase in the gene expression of CD204, CD206 and CD163 compared to M0-controls (p<0.01 for CD204 and CD206; p<0.05 for CD163) (Fig 5).

Moreover, ET-1 significantly induced the over-expression of IL-10 and CCL-22 compared to M0-controls (p<0.05 for both), showing effects similar to those induced by IL-4 (p<0.05 for CD204, CD163 and IL-10; p<0.01 for CD206 and CCL-22 vs. M0-controls) (Fig 5).

The effects of ET-1 were contrasted by treatment with ET_A/B_RA, which significantly antagonized the increased gene expression of CD204, CD206, CD163, IL-10 and CCL-22 (p<0.05 for CD204, CD206 and CD163; p<0.01 for IL-10 and CCL-22 vs. ET-1 treated cells) (Fig 5).

**Fig 6:**
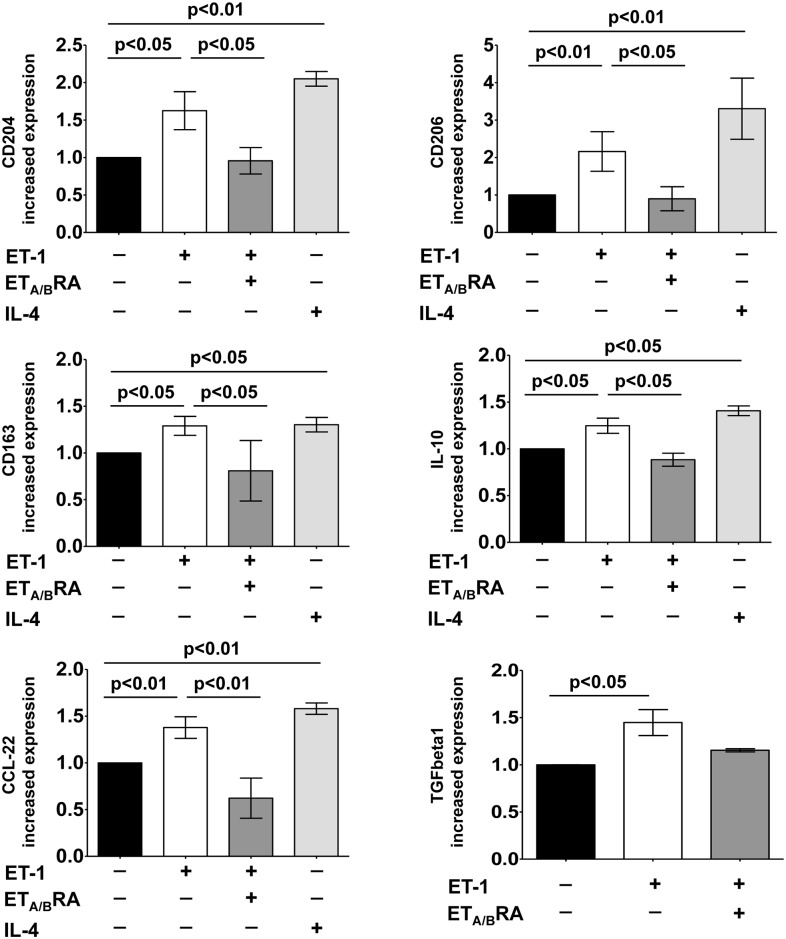
Evaluation of gene expression of M2 macrophage phenotype markers and TGFbeta1 in cultured human monocyte-derived macrophages. Quantitative real time polymerase chain reaction (qRT-PCR) of CD204, CD206, CD163, IL-10, CCL-22 and TGFbeta1 gene expression in cultured human monocyte-derived macrophages treated for 6 days with ET-1 (100nM) and IL-4 (10ng/mL) alone, or pre-treated with ET_A/B_RA (bosentan, 10^-5^M) for 1 hour before being stimulated with ET-1. Cultured human monocyte-derived macrophages maintained for 6 days in RPMI at 10% of FBS were used as untreated cells. The qRT-PCR was performed on six independent *in vitro* experiments and the data of CD204, CD206, CD163, IL-10, CCL-22 and TGFbeta1 gene expression are shown as mean±SD and indicated as increase in gene expression.

In primary cultures of monocyte-derived macrophages, ET-1 induced a significant increase in the gene expression of all the investigated M2 phenotype markers compared to untreated cells (p<0.05 for CD204, CD163 and IL-10; p<0.01 for CD206 and CCL-22) (Fig 6).

Also in primary cultures, the ability of ET-1 to increase the gene expression of all these M2 phenotype markers was shown to be similar to that determined by IL-4 (p<0.01 for CD204, CD206; CCL-22; p<0.05 for CD163 and IL-10 vs. untreated cells) (Fig 6).

In addition, ET-1 significantly up-regulated the gene expression of TGFbeta1 (p<0.05 vs. untreated cells) (Fig 6).

ET_A/B_RA significantly antagonized the ET-1-induced increase in the gene expression of M2 phenotype markers in these cultured cells (p<0.05 for CD204, CD206, CD163, IL-10; p<0.01 for CCL-22 vs. ET-1 treated cells) (Fig 6). Moreover, ET_A/B_RA treatment contrasted the ET-1-induced up-regulation of TGFbeta1 (non significantly) (Fig 6).

The effects of the combined treatment with ET-1 and IL-4 in inducing the expression of protein and gene expression of M2 macrophage phenotype markers were investigated and no synergistic effects on both cultured M0 and human monocyte-derived macrophages were observed (data not shown).

### Effects of ET-1, ET_A/B_RA and IL-4 on MMP-9 Production in Cultured Macrophages

**Fig 7:**
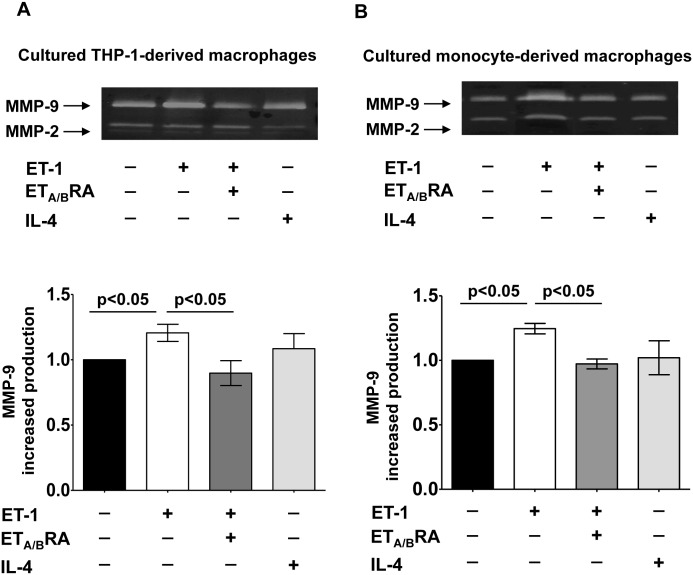
Evaluation of pro-fibrotic MMP-9 production in cultured macrophages. Evaluation by gel zymography of MMP-9 production and related densitometric analysis in cultured M0-macrophages and human monocyte-derived macrophages. (A) Cultured M0 macrophages were treated for 72 hours with ET-1 (100nM) and IL-4 (10ng/mL) alone, or pre-treated with ET_A/B_RA (bosentan, 10^-5^M) for 1 hour before the stimulation with ET-1. Cultured M0 macrophages maintained for 72 hours in RPMI at 5% of FBS were used as controls (M0-controls). (B) Cultured human monocyte-derived macrophages treated for 6 days with ET-1 (100nM) and IL-4 (10ng/mL) alone, or pre-treated with ET_A/B_RA (10^-5^M) for 1 hour before the stimulation with ET-1. Cultured human monocyte-derived macrophages maintained for 6 days in RPMI at 10% of FBS were used as untreated cells. Gel zymography was performed on six independent *in vitro* experiments and the data of MMP-9 production are shown as mean±SD.

In cultured M0 macrophages, ET-1 induced a significant production and release of MMP-9 compared to M0-controls (p<0.05) (Fig 7A).

The increased production of MMP-9 induced by ET-1 was significantly contrasted by treatment with ET_A/B_RA (p<0.05 vs. ET-1 treated cells) (Fig 7A).

Similar results were observed in primary cultures of human monocyte-derived macrophages (Fig 7B). In these cultured cells, ET-1 significantly increased the production and release of MMP-9 compared to untreated cells (p<0.05) (Fig 7B). The ET-1-mediated increase of MMP-9 was significantly contrasted by treatment with ET_A/B_RA (p<0.05 vs. ET-1 treated cells) (Fig 7B).

Conversely, IL-4 did not induce the over-production of MMP-9 in either cultured M0 macrophages or human monocyte-derived macrophages (Fig 7).

## Discussion

The results of the study show, for the first time, that ET-1 induces the over-expression of CD204, CD206 and CD163 in cultured human macrophages and therefore their transition into the M2 phenotype.

As known, M2 macrophages, together with the expression of these markers, share an IL-12^low^, IL-23^low^ and IL10^high^ phenotype and produce high levels of CCL-22 compared to M1 macrophages, which are IL-12^high^, IL-23^high^ and IL10^low^ cells [1–3].

The ability of ET-1 to induce the up-regulation of IL-10 and CCL-22 in cultured human macrophages further suggests its possible action as an inducer of the M2 phenotype. Of note, the effects of ET-1 in up-regulating all these M2 markers were found contrasted by the blockage of ET_A_ and ET_B_ receptors through the action of the dual ET receptor antagonist bosentan, suggesting a possible receptor-dependent mechanism for ET-1 in inducing the M2 phenotype in cultured human macrophages.

The value of present results lies also in the fact that they were obtained in cultured macrophages derived from the differentiation of both THP-1 cells and human circulating monocytes.

As recently discovered, M2 macrophage polarization is involved in the pathogenesis of several fibrotic diseases, including pulmonary fibrosis and SSc [9,26]. In idiopathic pulmonary fibrosis (IPF), the over-expression and activation of CD204 in alveolar macrophages shifted these cells to a pro-fibrotic M2 phenotype [26]. Moreover, in mouse models of pulmonary fibrosis, the blockage of CD204 (by the action of a specific blocking monoclonal antibody) in M2 macrophages was shown to completely abrogate the development of fibrotic process [26–28].

In SSc patients, a significant portion of circulating cells belonging to the monocyte/macrophage lineage abnormally differentiates into a CD14^bright^CD204^+^CD163^+^ cell subset. This latter characterizes the immune inflammatory infiltrate of the skin, indicating a possible contribution of these cells in the pathogenesis of the SSc fibrosis [9,10]. The presence of CD163^+^macrophages was also observed in acute and chronic inflammation, in wound healing sites of SSc patients (with interstitial lung disease) and in a number of pathological conditions, including pulmonary fibrosis [29–31]. In addition to that, the expression of CD206 was highly increased in SSc patients with pulmonary arterial hypertension (PAH), correlating with pulmonary artery pressure and PAH-related mortality [32]. Finally, the CD206^+^M2 macrophages may trigger the activation of fibroblasts through the over-production of IL-10 and CCL-22, contributing to the fibrotic progression in patients with IPF [31,33].

The results of the study show that the ET-1-induced M2 polarization seems to be similar to that mediated by IL-4, which represents an important M2 inducer [1–4].

As known, IL-4 induces macrophages to polarize primarily into the M2a phenotype, which is involved in the scar tissue formation in both wound healing and fibrosis [34]. Moreover, M2a macrophages are found in several diseases characterized by an extensive fibrosis [35,36]. Interestingly, increased serum levels of IL-4 have been detected in SSc patients, suggesting how this cytokine, by activating of M2 macrophages, might be involved in the pathogenesis of the disease [33,37].

The ability of ET_A/B_RA to contrast the induction of the M2 phenotype might contribute to attenuate ET-1 effects in those diseases characterized by extensive fibrosis and macrophage infiltrate, such as SSc.

The presence of M2 macrophages was also observed in cancer tissues and correlates with a more malignant phenotype characterized by a higher tumour invasion, the presence of lymph node metastasis and a poor prognosis [38–40]. These cells are called tumour associated macrophages (TAMs) and are characterized by the expression of CD204, CD206, CD163 [41]. TAMs also express high levels of IL-10 and CCL-22, which are necessary for the recruitment of T-regulatory cells to the tumour site to maintain an immunosuppressive microenvironment [41–43]

Another important feature of M2 macrophages is to be a major source of pro-fibrotic TGFbeta1 as well as MMP-9, contributing both directly and indirectly to fibrotic processes in several diseases, including IPF and SSc [1,3,8,44].

In primary cultures of human monocyte-derived macrophages, the observed ability of ET-1 to significantly increase both gene and protein expression of TGFbeta1 and, unlike to IL-4, MMP-9 production might suggest its possible involvement in establishing the formation of a fibrotic environment.

As known, TGFbeta1 and MMP-9 are primarily produced by TAMs compared to the other macrophage phenotypes and seem to be necessary to promote the migration and invasion of tumour cells [45]. In addition, TGFbeta1 is responsible for the transition of endothelial and epithelial cells as well as fibroblasts into myofibroblasts, therefore further contributing to fibrosis [13,46].

Macrophage-released MMP-9 contributes to fibrosis by inducing the activation of myofibroblasts, playing an important role in the pathogenesis of diseases characterized by an inflammatory etiology and extracellular matrix remodelling that requires selected therapies [44,47–51].

The ability of ET_A/B_RA (bosentan) to contrast these ET-1 effects might be important primarily in those fibrotic diseases characterized by high circulating levels of ET-1 and by the presence of macrophage infiltrate, as observed in SSc and type 2 diabetes [11,12].

Since this study primarily analysed M2 markers (i.e. CD204, CD206, CD163 and IL-10) and molecules related to a fibrotic phenotype (i.e. CCL-22, TGFbeta1 and MMP-9), the evaluation of other cytokines and chemokines (i.e. IL-12, CCL-2, CCL-17, CCL-18) produced by M2 macrophages will be necessary, in order to better characterize the ET-1-induced M2 phenotype compared to other M2 sub-populations (i.e. M2b, M2c). At the same time, the intracellular signalling pathways responsible for the M2 polarization mediated by ET-1 should be investigated.

A limitation of the present research is that only the effects of a dual ET_A/B_RA, which acts on both ET_A_ and ET_B_ receptors, was investigated. Further experiments evaluating the blockage of ET_A_ and ET_B_ receptor singularly, through the action of a selective ET_A_ or ET_B_ receptor antagonist, should be done.

In conclusion, macrophages might appear to be a possible further cellular mediator of ET-1 effects through their transition into a “pro-fibrotic” M2 phenotype, which seems to share features and properties with M2a and TAM sub-populations.

## Supporting Information

S1 Fig Evaluation of the time-dependent effects of ET-1 on the gene expression of M2 macrophage phenotype markers in cultured THP-1-derived macrophages. Quantitative real time polymerase chain reaction (qRT-PCR) of CD204, CD206 and CD163 gene expression in cultured THP-1-derived macrophages (M0 macrophages) treated for 24, 48 and 72 hours with ET-1 (100nM). Cultured M0 macrophages maintained for 24, 48 and 72 hours in RPMI at 5% of FBS were used as controls (M0-controls). The qRT-PCR was performed on four independent in vitro experiments and the data of CD204, CD206 and CD163 gene expression are shown as mean±SD and indicated as increase in gene expression.
(TIF)
