# Correction: Alternatively Activated (M2) Macrophage Phenotype Is Inducible by Endothelin-1 in Cultured Human Macrophages

**DOI:** 10.1371/journal.pone.0175238

**Published:** 2017-03-30

**Authors:** Stefano Soldano, Carmen Pizzorni, Sabrina Paolino, Amelia Chiara Trombetta, Paola Montagna, Renata Brizzolara, Barbara Ruaro, Alberto Sulli, Maurizio Cutolo

Fig 1 has an incorrectly duplicated panel. The image for the IL-4 treated CD68 cells is an incorrect duplication of the CD68 ET-1 treated cells. Please see the correct Fig 1 here.

**Fig 1:**
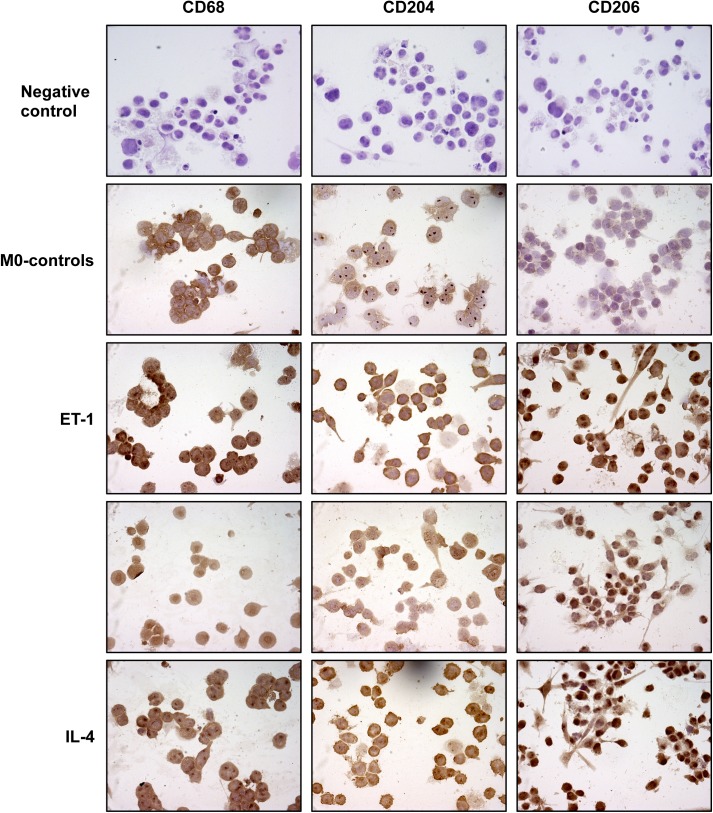
Evaluation of CD68, CD204 and CD206 expression in cultured THP-1-derived macrophages. Immunocytochemistry of CD68 (marker of macrophage activation), CD204 and CD206 protein expression in cultured THP-1-derived macrophages (M0 macrophages) treated for 72 hours with ET-1 (100nM) and IL-4 (10ng/mL) alone, or pre-treated with ET_A/B_RA (bosentan, 10^-5^M) for 1 hour before being stimulated with ET-1. Cultured M0 macrophages maintained for 72 hours in RPMI at 5% of FBS were used as controls (M0-controls). Immunocytochemistry was performed on four independent *in vitro* experiments. The detection of protein expression was performed evaluating the same number of cells by light microscopy (magnification 40X).
