# Supplementary figures and images for: Alternatively Activated (M2) Macrophage Phenotype Is Inducible by Endothelin-1 in Cultured Human Macrophages
Source: PLoS One. 2016 Nov 15;11(11):e0166433. doi: 10.1371/journal.pone.0166433 (PMC5112853; doi:10.1371/journal.pone.0166433)

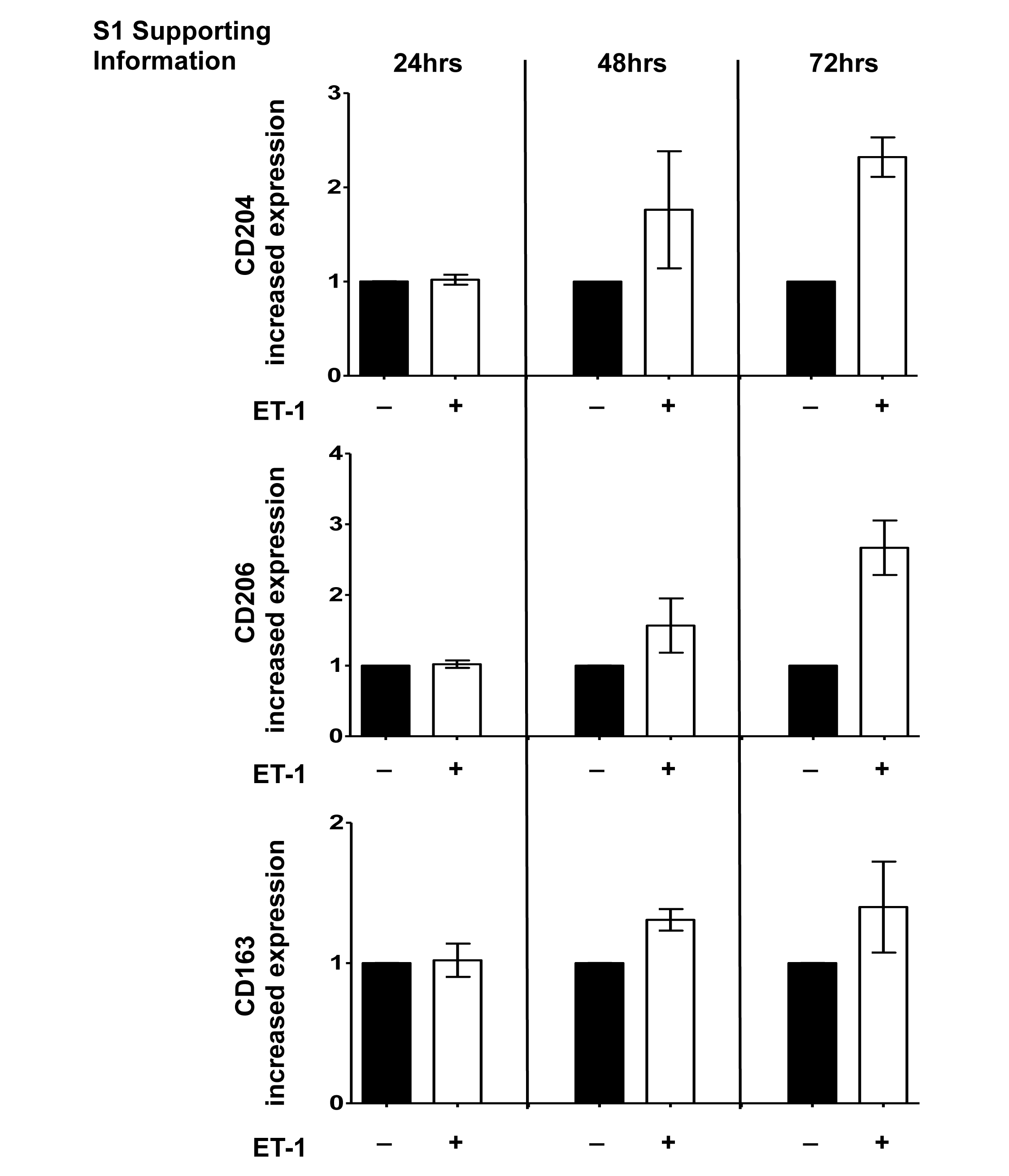

Supplement: S1 Fig — Quantitative real time polymerase chain reaction (qRT-PCR) of CD204, CD206 and CD163 gene expression in cultured THP-1-derived macrophages (M0 macrophages) treated for 24, 48 and 72 hours with ET-1 (100nM). Cultured M0 macrophages maintained for 24, 48 and 72 hours in RPMI at 5% of FBS were used as controls (M0-controls). The qRT-PCR was performed on four independent in vitro experiments and the data of CD204, CD206 and CD163 gene expression are shown as mean±SD and indicated as increase in gene expression. (TIF) [file pone.0166433.s001.tif]
